# Impact of differentiating between persistent and new infections on colposcopy referral in HPV-positive triage-negative women: results from the NTCC2 study

**DOI:** 10.1186/s13027-025-00713-8

**Published:** 2025-11-20

**Authors:** Annarosa Del Mistro, Pamela Mancuso, Francesca Carozzi, Laura De Marco, Simonetta Bisanzi, Giampaolo Pompeo, Guglielmo Ronco, Silvia Gori, Elena Allia, Daniela Gustinucci, Helena Frayle, Anna Iossa, Elena Cesarini, Simonetta Bulletti, Basilio Passamonti, Jessica Viti, Laura Toniolo, Francesco Venturelli, Paolo Giorgi Rossi, Maria Benevolo, the NTCC2 Working Group

**Affiliations:** 1 Istituto Oncologico Veneto IOV—IRCCS, Padua, Italy; 2 Epidemiology Unit, Azienda Unità Sanitaria Locale—IRCCS di Reggio Emilia, via Amendola 2, 42122 Reggio Emilia, Italy; 3 Institute for Cancer Research, Prevention and Oncological Network (ISPRO), Florence, Italy; 4 Centre for Cervical Cancer Screening, City of Health and Science Hospital, Turin, Italy; 5 Unit of Cancer Epidemiology and Centre for Cancer Prevention (CPO), City of Health and Science Hospital, Turin, Italy; 6 Laboratorio Unico di Screening, USL Umbria 1, Perugia, Italy; 7 ULSS6 Euganea, Padua, Italy; 8 IRCCS—Regina Elena National Cancer Institute, Rome, Italy

**Keywords:** Cervical cancer screening, Human papillomavirus, Genotyping, HPV persistence, HPV new infections, Cervical intraepithelial neoplasia

## Abstract

**Background:**

In cervical screening, human papillomavirus (HPV)-positive women at 1-year retesting are typically referred to colposcopy. This study, by the use of extended genotyping, estimates the impact of distinguishing persistent from new infections in an effort to reduce colposcopy referral. It also evaluates whether the new infection rate varies according to genotyping, cytology, p16/ki67, and E6/E7 mRNA results.

**Methods:**

We analyzed data from HPV-DNA-positive women at baseline in the NTCC2 trial genotyped with the Onclarity HPV assay. Eligible participants were Onclarity-positive women without baseline CIN2+ who had a cervicovaginal sample collected at least 10 months after baseline. Persistent infections were defined as cases with at least one common genotype between baseline and follow-up specimens. New infections were defined as cases positive for different genotypes at follow-up, with no baseline genotypes detected.

**Results:**

Among 1,540 women included, 613 were Onclarity-positive at both baseline and 1-year retesting: 488 (79.6%) had persistent infections, while 68 (11.1%) had new ones. All the 11 CIN3 cases identified at follow-up occurred in women with persistent infections. The new infection rate was significantly higher in women with baseline single-channel compared to multiple channels positivity (13.3% *vs* 5.5%, p=0.001). Stratifying by age, persistence and new infection rates were significantly different among different age groups (respectively p=0.005 and p=0.009). No significant difference in both new infections and persistence rate was observed between baseline cytology positive and negative cases. Stratifying by baseline p16/ki67 results, we found a significantly higher rate of persistent cases among p16/ki67 positive cases and of new infections among p16/ki67 negative cases (respectively p=0.013 and p=0.016). E6/E7 mRNA findings affected only persistence rate showing a significant difference in the proportion of persistent cases between positive and negative cases (p=0.004), but did not affect new infection rate.

**Conclusion:**

Extended genotyping classified 11.5% of 1-year HPV-positive cases as new infections. As 1-year HPV positivity accounts for about 60% of first-level colposcopies, this corresponds to about 7% of colposcopies. Baseline single-channel positivity, age, and p16/ki67-negative results may influence this proportion.

**Trial registration:**

Clinicaltrials.gov registration number: NCT01837693, New Technology in Cervical Cancer 2 (NTCC2) study.

**Supplementary information:**

The online version contains supplementary material available at 10.1186/s13027-025-00713-8.

## Background

Human papillomavirus (HPV)-based screening is now replacing cytology-based screening to detect precancerous cervical lesions. This approach requires strategies to triage HPV-positive women, distinguishing those who need immediate colposcopy from those suitable for retesting [1–3].

To stratify HPV-positive women, several biomarkers have been proposed [4–8]. Useful information for risk stratification can also be obtained from the longitudinal history of the infection. HPV persistence is a major risk factor for developing high-grade cervical lesions [9–12]. Notably, HPV16 persistence is associated with the highest absolute risk of progression to cancer, a risk significantly higher than that of other high-risk HPV types [13, 14]. Furthermore, women treated for cervical intraepithelial neoplasia grade 2 or worse (CIN2+) who experience, during follow up, a genotype switch and clear the original infection, rarely develop new CIN2 or CIN3 lesions [12, 15, 16]. Therefore, distinguishing persistent from new infections in women retested for HPV may help optimize management strategies.

The Italian screening protocol, similar to the Multi Societal US guidelines and those of many other countries [17, 18], recommends cytology as the triage test for HPV-positive women and refers baseline HPV-positive/cytology-negative women to 1-year HPV retesting. More than 50% of them remain positive after 1 year and are referred to colposcopy [4, 19]. However, some of these women may have cleared the baseline infection and acquired a new one [12]. These women have a lower risk of cervical intraepithelial neoplasia grade 3 or worse (CIN3+) and a lower short-term probability of developing cancer than women with persistent HPV infections [9, 20]. Thus, there is a rationale for referring to colposcopy only women with persistent infections, and managing women with new infections differently, for example retesting them after further 12 months.

Similarly, in the follow-up of baseline HPV-positive/triage-positive women, most guidelines recommend [17, 18] that those who underwent immediate colposcopy but were negative for CIN2+ lesions repeat HPV testing after 1-year. In this scenario, the management of women with new infections could differ from that of those with persistent infections at retesting.

By using cervical cell samples stored in the New Technologies for Cervical Cancer 2 (NTCC2) biobank, we genotyped all the HPV-DNA positive samples using a commercially available HPV screening assay that provides extended genotyping [21, 22]. Our aim was to assess, among women still HPV-positive at 1-year retesting, the proportion of cases attributable solely to new infections compared with those with persistent infections. This estimate was reported for both groups: triage-positive and triage-negative women.

This information provides a basis for estimating the potential impact of distinguishing new from persistent infections on overall colposcopy referral within the screening algorithm. Furthermore, the study evaluated whether the proportion of persistent and new infections varied according to baseline extended genotyping, age, cytology, p16/ki67 dual staining and E6/E7 HPV mRNA results.

## Methods

### NTCC2 study design

The NTCC2 study design and main results have been previously published [4]. Briefly, 41,127 women aged 25–59 years were recruited from five Italian HPV-DNA-based cervical cancer screening centers. Cervical samples were collected in PreservCyt solution (Thin Prep, Hologic), and HPV-DNA results were obtained using either the Hybrid Capture (HC2; QIAGEN) or the Cobas 4800 (Roche Diagnostics) HPV test (hereinafter referred as to Cobas/HC2). All baseline HPV-DNA-positive women were triaged using cervical cytology. According to the NTCC2 study protocol, these women were also tested for p16/ki67 dual staining using the CINtec PLUS assay (Roche Diagnostics) and for E6/E7 HPV mRNA overexpression using the APTIMA assay (Hologic). Cytology, p16/ki67, and E6/E7 mRNA testing methods and their interpretation were described elsewhere [4]. In addition, a 2 mL aliquot of the cervicovaginal sample was stored at −80 °C in a dedicated biobank for each HPV-positive woman.

Using the atypical squamous cells of undetermined significance (ASC-US) threshold, women positive at cytology triage were referred for immediate colposcopy, with punch biopsies of abnormal areas only. If colposcopy results were negative for CIN2+ lesions, the women were referred for 1-year HPV retesting. Cytology-negative women were randomized into two arms: immediate colposcopy or repeat HPV-DNA testing after 1 year. Women who were still HPV-positive at 1-year retesting were referred for colposcopy.

### Study population

In the present analyses, we included only HPV-DNA-positive women who did not have CIN2+ lesions at baseline colposcopy and had two consecutive Cobas/HC2 samples: one at baseline and another collected at least 10 months after baseline but no later than 24 months. Most women had the second specimen taken after 12–13 months from baseline. All baseline samples were genotyped and women negative for the Onclarity assay at baseline were excluded from the analyses. Among the 1-year retested samples, those still positive for Cobas/HC2 were genotyped, while Cobas/HC2-negative samples were not genotyped and considered as cleared infections. Women were managed only based on the Cobas/HC2 results following the NTCC2 study design.

### Extended genotyping

As previously described [23], extended genotyping was performed on cervical samples stored in the NTCC2 biobank, utilizing the Onclarity HPV Assay (Becton & Dickinson). This is a real-time multiplex PCR-based assay targeting the E6/E7 region of the HPV genome. The assay detects 14 high-risk types, divided into nine channels, providing individual results for genotypes 16, 18, 45, 31, 51, and 52 and pooled results for genotypes 33/58, 35/39/68, and 56/59/66. HPV positivity can be identified in a single channel or simultaneously across multiple channels (multichannel).

### Statistical analyses

#### Outcome definition

The viral outcomes were defined based on the Cobas/HC2 and Onclarity results at retesting. Specifically, we categorized outcomes as follows:

1. Persistent infections: cases Cobas/HC2 positive and Onclarity positive for at least one of the channels detected at baseline, with or without additional positivity;
2. New infections: cases Cobas/HC2 positive and Onclarity negative for all the channels detected at baseline and positive for new channel(s) not detected at baseline;
3. Negative for typing: cases Cobas/HC2 positive and Onclarity negative, i.e., no positive channels detected at the second test;
4. Negative for Cobas/HC2: these samples were not typed and considered as cleared;
5. Total cleared: the sum of cases 2, 3, and 4.

The main endpoint was the proportion of women positive only for new infections among those positive for at least one channel at follow-up. We conducted a proportional analysis among women to examine the distribution of the outcomes according to multiple infections, age and baseline cytology, p16/ki67, and E6/E7 mRNA results. Logistic regression models were used to estimate the probability of these outcomes, in terms of odds ratios (ORs). We reported predicted proportions adjusted for age, using marginal standardization methods to account for potential age-related confounding. We did not adopt a threshold for rejecting the null hypothesis, because the study had been sized for different outcomes and questions, thus a formal statistical test of hypothesis would not be meaningful, particularly when the null hypothesis is not rejected. The reported confidence intervals should be considered as a measure of the precision of the estimates, and p-values should be interpreted as continuous variables.

To evaluate the distribution of Onclarity channel positivity in samples with multichannel positivity, we considered the following:

1. Persistence of positivity for the same channel: cases positive for the same channel at baseline and at retesting, independently from the positivity of other channel(s) at either timepoint;
2. Positivity for other channel(s): cases that were positive at retesting for one or more channels resulted negative at baseline and became negative for channels that were positive at baseline.

To compute the total clearance, the reported percentage was adjusted to account for samples for which genotyping at follow-up was unavailable.

We computed the proportion of colposcopies that could be avoided in women with only new infection, that is, the proportion of only new infection cases multiplied by the proportion of colposcopies generated by the 1-year retesting on total colposcopy referral (baseline + 1-year in our study).

## Results

### Description of population under study

Supplementary Figure 1 presents the study flow chart. All the 3,129 baseline Cobas/HC2-positive cases (*n* = 1,436 tested with Cobas 4800 and *n* = 1,693 with HC2) were genotyped. Among these, 764 were Onclarity negative and were excluded from the analyses. The cytological triage of the 2,365 Cobas/HC2 and Onclarity-positive cases showed 2,334 cases with a valid report (690 ASC-US+ and 1,644 NILM) and 31 with an inadequate or missing report. 1,540 of 2,365, including 19 cases with inadequate or missing cytology, underwent a second HPV test at 1-year retesting (between 10 and 24 months after baseline, median 13.5 months), and were included in the study. Among the 1,540 cases, we identified 23 CIN2+ lesions, of which 11 were CIN3+. Onclarity typing results stratified by the baseline HPV-DNA assay are shown in Supplementary Table 1.

### Overall distribution of persistence and new infections

Table 1 summarizes the distribution of persistence and new infections of baseline infections, based on Cobas/HC2 and Onclarity baseline and 1-year retesting results. Most of the samples (1,219, 79.1%) were positive for a single channel at baseline. At retesting, 61.8% (951/1,540) remained positive for Cobas/HC2, and 613 of these were genotyped. Among the genotyped samples, the majority were still positive for at least one channel in common with baseline (488/613, 79.6%), and all 11 CIN3 lesions were diagnosed within this group. Conversely, 68 samples (11.1%) were positive for different channel(s) compared with baseline. Furthermore, 57 (9.3%) resulted negative for the Onclarity test, including 7 CIN2+, 3 of them CIN3, representing a lack of sensitivity of the Onclarity assay, as previously reported by our group^23^. Regarding the CIN2+ and CIN3 lesion distribution (Table 1 and Suppl. Table 2), no CIN3 cases were identified in the group with new infections, where only CIN2+ lesions were diagnosed. The proportion of CIN2+ was similar in women with persistent and new infections (20/488 and 3/68, 4.1% vs 4.4%, respectively).

**Table 1:** Distribution of persistence and new infections according to Onclarity results in 1,540 baseline Cobas/HC2 and Onclarity-positive samples from women without CIN2+ at baseline and with a second HPV test 1 year after baseline

	Baseline Onclarity positive	FU negative for Cobas/HC2 HPV	Typing of follow-up positive for Cobas/HC2 HPV samples						
			FU Positive for Cobas/HC2 HPV	Samples with typing					Missing typing
				Persistence		Only new infections		Negative for typing	
	*n*	*n*	*n*	*n*	n of CIN3+*	*n*	n of CIN3+*	*n*	*n*
Single channel	1,219	509	710	345	*10*	59	*0*	44	262
Multichannel	321	80	241	143	*1*	9	*0*	13	76
**Total**	**1,540**	**589**	**951**	**488**	*11*	**68**	*0*	**57**	**338**

* Among the CIN3+ cases, no cancer was diagnosed

### Persistence, new infections, and total clearance according to age, baseline cytology, p16/ki67, and E6/E7 mRNA results

To evaluate whether the number of positive channels, age distribution, baseline cytology, p16/ki67, and E6/E7 mRNA results affect persistence of HPV infection, indeed affecting the proportion of new infections, we stratified the genotyping results according to these findings, including only the cases with a valuable report (Suppl. Table 3) and adjusting these findings by age (Table 2). Persistence was significantly higher among cases with multichannel positivity (86.9% vs 76.9%, *p* = 0.002) whereas the proportion of new infections was significantly higher among cases with single-channel positivity (13.3% vs 5.5%, *p* = 0.001).

**Table 2:** Adjusted predicted marginal proportions of persistence, new infections, and total clearance based on multiple infections, age, cytology, p16/ki67, and E6/E7 mRNA results

	Baseline Onclarity positive	FU negative for Cobas/HC2 HPV	Typing of follow-up positive for Cobas/HC2 HPV samples				Total clearance of baseline infections^#^
			FU Positive for Cobas/HC2 HPV	Samples with typing			
				Persistence	Only new infections	Negative for typing	
	n	% on total samples (a)	% on total samples	% on positive and typed	% on positive and typed (b)	% on positive and typed (c)	%
**Total**	**1,540**	**38.3**	**61.7**	**79.4**	**11.3**	**9.3**	**51.0**
**Multiple infections**							
Single channel	1,219	41.9	58.1	76.9	13.3	9.8	55.3
Multichannel	321	24.6	75.4	86.9	5.5	7.8	34.6
**Age**							
< 40	447	39.8	60.2	72.0	16.5	11.5	56.7
40–50	670	39.6	60.4	83.3	9.3	7.4	49.7
> 50	423	34.5	65.5	83.3	7.1	9.6	45.4
Cytology*							
Negative	1,163	39.3	60.7	80.9	10.8	8.3	50.9
Positive	358	34.8	65.2	75.5	12.3	12.3	50.8
E6/E7 mRNA*							
Negative	295	48.1	51.9	67.2	13.7	19.1	65.1
Positive	1,243	36.0	64.0	82.0	10.5	7.5	47.5
p16/ki67*							
Negative	991	41.1	58.9	76.9	13.8	9.3	54.7
Positive	437	28.2	71.8	84.9	7.3	7.9	39.0

# Total clearance includes women who at 1-year retesting were negative for Cobas/HC2 and those who tested negative for Onclarity for the channels that were positive at baseline: a+(1-a)*(b+c)

* Only samples with a valid test result were included: 19 missing or inadequate cytology; 2 missing E6/E7 mRNA; 112 missing or non-valuable p16/ki67

Regarding age distribution, we observed a significant difference in both persistence and new infection rate among different age groups (respectively *p* = 0.005 and *p* = 0.009).

Baseline cytology results revealed no significant difference in the rate of new infections and of persistent cases between cytology positive and negative cases. When dividing the cytology positive reports into Low-Grade (ASC-US and L-SIL) and High-Grade (AGC, ASC-H, HSIL, AIS) categories, we similarly observed no significant differences (data not shown).

Stratifying by baseline p16/ki67 results, we found a significantly higher rate of persistent cases among p16/ki67 positive cases and of new infections among p16/ki67 negative cases (respectively *p* = 0.013 and *p* = 0.016). Stratification by E6/E7 mRNA findings showed a significant difference in the proportion of persistent cases between positive and negative cases (82.0% vs 67.2%, *p* = 0.004) but did not affect the proportion of new infections.

When we analyzed the above-mentioned results considering the number of positive channels, we observed that the persistence was higher in multichannel cases in all strata (Suppl. Table 3).

Total clearance was 51.0%, being higher among samples with single-channel positivity compared to multichannel positivity (55.3% vs 34.6%). The highest total clearance was observed among E6/E7 mRNA negative samples (65.1%), while the lowest was among p16/ki67 positive samples (39.0%).

Moreover, no significant differences were observed in CIN2+ distribution between persistent and new infections, even when stratified by cytology, p16/ki67, or E6/E7 mRNA results (Suppl. Table 2).

### Persistence, new infections and total clearance according to channel positivity

We analyzed the distribution of persistence, new infections, and clearance for each channel separately and considered single and multichannel positivity independently (Table 3). While the proportion of new infections was similar across different genotypes (Table 3A), we found that the behavior of each channel differed depending on whether it was positive as a single channel or in a multichannel context (Table 3B). For example, HPV16 was more persistent and had twice the rate of new infections when present as a single infection, whereas HPV51 showed higher clearance in single-channel cases than in multichannel cases. However, all these differences could result from random fluctuations due to the small sample size.

**Table 3:** Persistence, new infections, and total clearance by baseline channel(**s**) in single (**A**) or multichannel (**B**) context

	Baseline Onclarity positive	FU negative for Cobas/HC2 HPV	Typing of FU positive for Cobas/HC2 HPV samples						Total clearance of baseline infections^**#**^
			FU positive for Cobas/HC2 HPV	Samples with typing				Missing typing	
				Persistence	Persistence other types	Only new infections	Negative for typing		
	n	n (% on total samples - a)	n (% on total samples)	n (% on positive and typed)	n (% on positive and typed)	n (% on positive and typed - b)	n (% on positive and typed - c)	n	%
**A) Single channel**									
16	196	58 (29.6)	138 (70.4)	70 (84.3)		9 (10.8)	4 (4.8)	55	40.6
18	65	19 (29.2)	46 (70.8)	16 (72.7)		4 (18.2)	2 (9.1)	24	48.5
45	50	23 (46)	27 (54)	15 (88.2)		2 (11.8)	0 (0)	10	52.4
33/58	129	52 (40.3)	77 (59.7)	32 (78)		3 (7.3)	6 (14.6)	36	53.4
31	176	56 (31.8)	120 (68.2)	66 (78.6)		11 (13.1)	7 (8.3)	36	46.4
56/59/66	271	124 (45.8)	147 (54.2)	75 (78.1)		13 (13.5)	8 (8.3)	51	57.6
51	96	55 (57.3)	41 (42.7)	19 (67.9)		3 (10.7)	6 (21.4)	13	71
52	95	44 (46.3)	51 (53.7)	25 (71.4)		7 (20)	3 (8.6)	16	61.7
35/39/68	141	78 (55.3)	63 (44.7)	27 (64.3)		7 (16.7)	8 (19)	21	71.3
**B) Multichannel**									
16	126	31 (24.6)	95 (75.4)	40 (66.7)	13 (21.7)	3 (5)	4 (6.7)	35	33.4
18	37	9 (24.3)	28 (75.7)	7 (35)	9 (45)	1 (5)	3 (15)	8	39.5
45	30	8 (26.7)	22 (73.3)	7 (41.2)	7 (41.2)	1 (5.9)	2 (11.8)	5	39.6
33/58	75	15 (20)	60 (80)	25 (59.5)	15 (35.7)	1 (2.4)	1 (2.4)	18	23.8
31	83	12 (14.5)	71 (85.5)	31 (62)	13 (26)	1 (2)	5 (10)	21	24.7
56/59/66	138	30 (21.7)	108 (78.3)	42 (56)	22 (29.3)	7 (9.3)	4 (5.3)	33	33.2
51	58	20 (34.5)	38 (65.5)	7 (29.2)	13 (54.2)	3 (12.5)	1 (4.2)	14	45.4
52	67	15 (22.4)	52 (77.6)	18 (51.4)	10 (28.6)	3 (8.6)	4 (11.4)	17	37.9
35/39/68	105	27 (25.7)	78 (74.3)	24 (43.6)	26 (47.3)	2 (3.6)	3 (5.5)	23	32.5

# Total clearance includes women who at 1-year retesting were negative for Cobas/HC2 and those who tested negative for Onclarity for the channels that were positive at baseline: a+(1-a)*(b+c)

### Impact of the new infection rate on women’s management

**Fig. 1:**
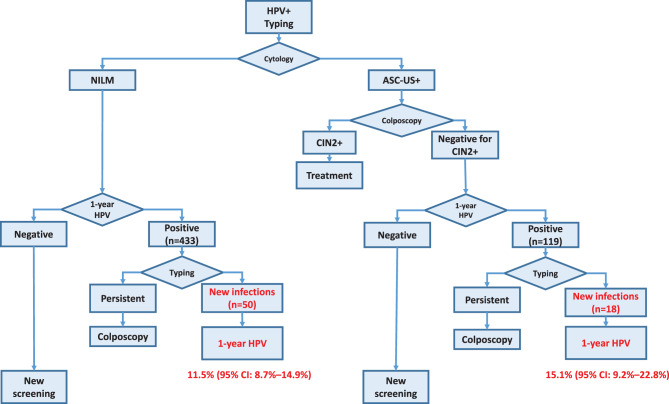
Proportion of HPV-positive women who presented new infections at 1-year retesting stratified by cytological triage

Overall, our results showed that the use of extended genotyping for samples HPV positive at baseline and at 1-year retesting allowed to differentiate between persistent and new infections (Fig. 1). In particular, among women retested because of HPV-positive/cytology-positive triage but colposcopy negative for CIN2+, 15.1% (95% CI: 9.2%–22.8%) were identified as having only new infections, while among women retested after HPV-positive/cytology-negative triage, 11.5% (95% CI: 8.7%–14.9%) were classified as new infections. Considering that in the Italian screening protocol, positivity at 1-year retesting of HPV-positive/cytology-negative women accounts for about 60% of colposcopy referral [19], identifying the group of those with new infections (11.5% in our study) could reduce the total colposcopy referral rate by about 7%.

## Discussion

### Main findings

By applying extended genotyping, we identified 11.5% of cases still HPV-positive at retesting as new infections. This proportion varied significantly by baseline Onclarity channel positivity, age, and baseline p16/ki67 results but was only slightly influenced by baseline cytology and E6/E7 mRNA findings.

No CIN3 were found among women with new infections. However, the study was underpowered to assess CIN3+ risk. In fact, based on the prevalence observed in the persistent group, the expected number of CIN3+ cases in the new infection group was very low, only 1.5. Furthermore, the prevalence of CIN2+ lesions was nearly identical between persistent and new infections.

### Limits

The main limitation of this analysis is that the typing test was not used as the primary screening test. Consequently, this may impact the results, as typing only the cases positive for another test increases overall specificity. In this study, we did not include cases that were Onclarity-positive but Cobas- or HC2-negative, which would be included among positive cases if Onclarity were used as the first or sole screening test. Women who are Onclarity-positive but Cobas- or HC2-negative would likely have a lower probability of persistent infections, similarly to what observed for women with discordant HPV results, i.e. Cobas- or HC2-positive and Onclarity-negative, who showed a high proportion of negative retesting [24]. However, we assumed that the probability of new infections was similar. As a result, we may have underestimated the proportion of new infections that could be observed in a screening program adopting genotyping as the primary screening test. Nevertheless, we quantified the proportion of women who are Onclarity-positive but Cobas or HC2-negative [23] as 1.5% of the sample that would approximately correspond to 19% of all Onclarity HPV-positive cases in the population.

Another limit is that we did not genotype about a third of the women who were Cobas/HC2 positive at 1-year retesting, because the samples were not stored in the biobank or because of insufficient residual material. Nevertheless, all the analyses were adjusted to account for the samples in which genotyping at retesting was unavailable.

Our results are not suitable for evaluating the natural history of single-genotype infections, as we did not include cytology-positive and p16/ki67-positive women with colposcopy positive for CIN2+, who were subsequently treated. As these women are very likely to have persistent HPV infections, their exclusion may result in a possible overestimation of new infections in an untreated population. However, our findings reliably represent women retested according to current screening protocols in most countries and, therefore, could be valuable in clinical practice. Additionally, the genotyping assay used in this study, the Onclarity assay, groups certain genotypes rather than detecting them individually, which limits the ability to specifically evaluate persistence. This grouping of genotypes reduces the capacity to discriminate between clearance and new infections. Many studies have reported new infection rates ranging from 7% to 60%. However, these results are difficult to compare because of variations in persistence definition, retesting intervals, study population, and the genotyping test employed [12].

### Implication for practice and research

Based on our understanding of the natural history of the disease [25], women with only new infections could be managed differently from those with persistent infections, potentially avoiding immediate colposcopy. The proportion of CIN2+ was similar in women with persistent and new infections, however, the absence of CIN3 cases, which are a better surrogate endpoint for precancerous lesions, in the new infection group, provides reassurance about the feasibility of not referring them to colposcopy and managing them differently.

Current US [18] and European guidelines [17] already suggest a different management for a newly detected infection and for infections that are persistently positive after one year. According to these guidelines, if a woman tests HPV-positive/cytology-negative at a new screening round should be referred to one-year retesting, and if the woman tests still HPV positive after one year, she would be referred to colposcopy. Therefore, for women who only have a new infection, the current recommendations could be interpreted in two ways: should these women be considered as “still HPV-positive” and referred to colposcopy or should they be considered a newly detected infection and triaged to decide about immediate colposcopy? We do not want to say which is the authentic interpretation; in this work, we only quantified the difference in terms of colposcopy workload between the two interpretations.

For baseline HPV-positive/cytology-negative women, this approach could reduce total referral to colposcopy by about 7%. This reduction is comparable to that achievable with the triage test combination now proposed in the literature, which, in this population, ranged from 2% to 17% [24]. Nevertheless, some of these women may still need colposcopy during subsequent follow-up.

In protocols with longer intervals for HPV-positive/triage-negative women, such as those proposed by the Swedish [26] and Dutch [27] guidelines and the new Italian recommendations [28], in which women can be referred to 3- or 5-year retesting without colposcopy, distinguishing persistent infections could even be more useful. With longer intervals, the proportion of new infections is expected to increase, and the number of women who could be managed differently would rise. On the other hand, after a longer follow-up, new infections may have persisted for a relatively long time. Nevertheless, the differential in the underlying risk between a new and a persistent infection would remain substantial, even after 3 or 5 years [29–31].

The impact on the follow-up of HPV-positive/triage-positive women after a negative colposcopy is more difficult to assess. In the follow-up of women who underwent colposcopy for an HPV-positive result and low-grade cytology, those still HPV-positive after 1 year and triaged with cytology should undergo further 1-year retesting if cytology is negative for intraepithelial lesion or malignancy or remains low-grade, according to the Italian guidelines and the “equal risk-equal management” principle adopted by the US guidelines. Applying the standard protocol for a new infection – cytology triage with colposcopy referral for positive results – could paradoxically result in more intensive management for a new infection than a persistent one. Therefore, although theoretically useful, the application of genotyping to distinguish persistent and new infections would have little, if any, practical impact on women’s management with the screening protocol in use. In the context of the “equal risk-equal management” approach, we should aim to assess the 5-year CIN3+ risk in women with new infections following a colposcopy negative for CIN2+. This would require very large studies with detailed cytological and molecular data. It is worth considering whether the risk in such a small group could instead be estimated using a priori knowledge of the underlying molecular mechanisms and pathogenesis and evidence from large cohort studies [9, 20] on the risks associated with new and persistent infections, rather than relying solely on empirical measurements.

Finally, the decision to incorporate type-specific persistence into management protocols should also take into account the increase in complexity that it introduces in the management algorithm. Probably the complexity of the current algorithms is already very high, and risk calculators should be implemented to support clinicians as well as organised screening programs to appropriately define the woman’s management at each step.

## Conclusion

The use of extended genotyping to distinguish new from persistent infections has the potential to reduce the colposcopy burden. Nevertheless, without appropriate guidelines for managing women with new infections, the practical utility of this approach may be limited.

## Electronic supplementary material

Below is the link to the electronic supplementary material.

Supplementary Material 1

Supplementary Material 2

## Data Availability

Individual participant data that underlie the results reported in this article, after deidentification, are available for investigators whose proposed use of the data has been approved by the S. Giovanni Battista University Hospital Ethic Committee, Turin, Italy, to achieve aims in the approved proposal. Proposals should be directed to paolo.giorgirossi@ausl.re.it and to comitatoetico@cittadellasalute.to.it. To gain access, data requestors will need to sign a data access agreement. The study protocol is freely available online.

## Abbreviations

HPV: Human papillomavirus
NTCC2: New Technologies for Cervical Cancer 2
ASC-US+: Atypical squamous cells of undetermined significance or a more severe report
CI: Confidence interval
CIN2+: Cervical intraepithelial neoplasia grade 2 or worse
CIN3+: Cervical intraepithelial neoplasia grade 3 or worse
NILM: Negative for intraepithelial lesions or malignancy
