# Supplementary material for: Impact of differentiating between persistent and new infections on colposcopy referral in HPV-positive triage-negative women: results from the NTCC2 study
Source: Infect Agent Cancer. 2025 Nov 20;20:84. doi: 10.1186/s13027-025-00713-8 (PMC12632028; doi:10.1186/s13027-025-00713-8)

Supplementary Figure 1: Flow chart of the 3,129 baseline HPV-DNA-positive women undergoing cytology triage, including cases with an inadequate or missing cytology result. As for the NTCC2 study design, cytology-positive women were referred to immediate colposcopy, while cytology-negative women were randomized to immediate colposcopy or 1-year HPV-DNA retesting. Women still HPV-DNA positive were referred to colposcopy. The outcome distribution is marked in red. CIN2+ and CIN3 lesions are reported stratified by outcome.

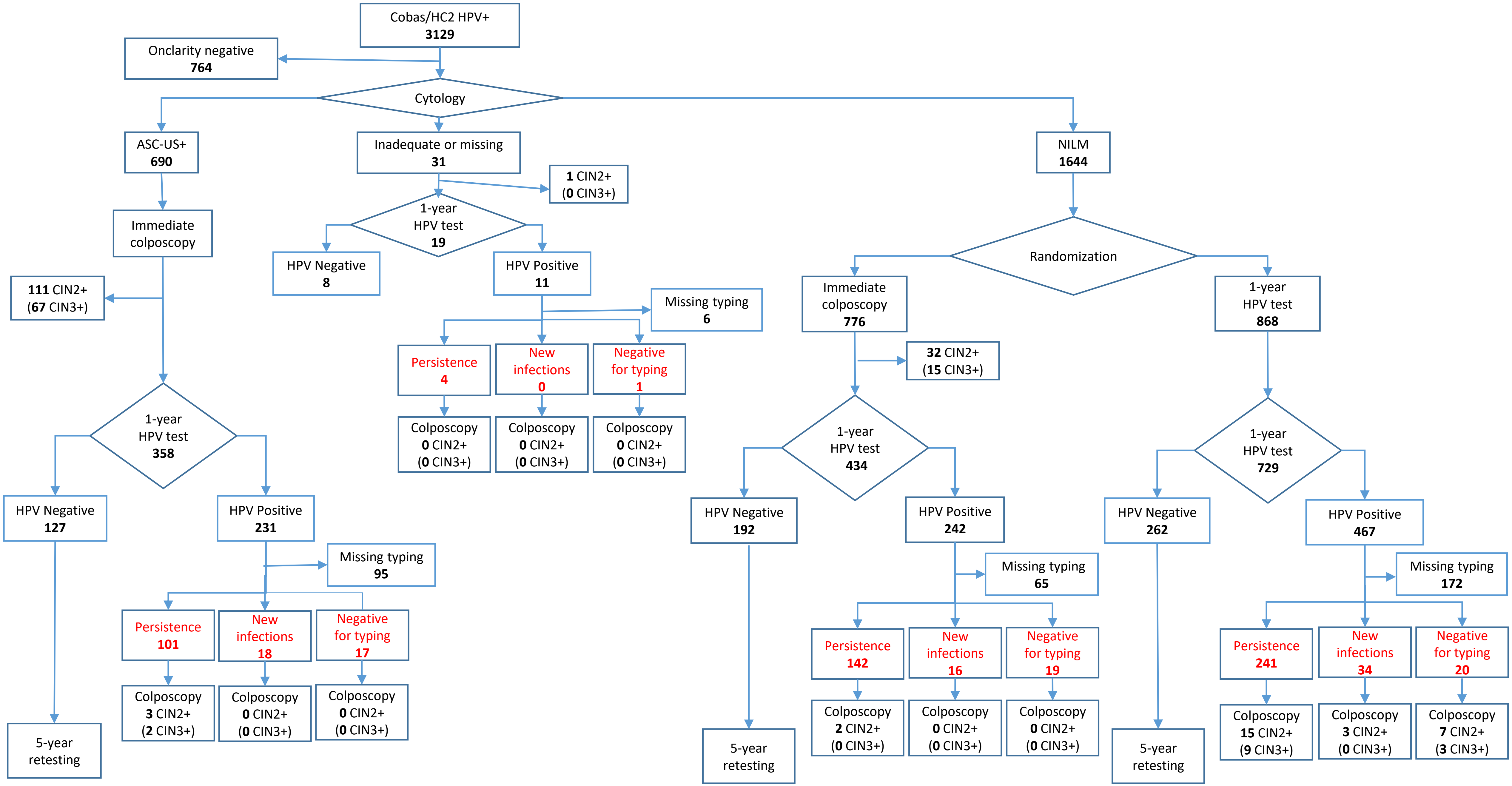

Supplement: Supplementary file 1 — Supplementary Material 1 [file 13027_2025_713_MOESM1_ESM.pdf]
