# Supplementary material for: Impact of differentiating between persistent and new infections on colposcopy referral in HPV-positive triage-negative women: results from the NTCC2 study
Source: Infect Agent Cancer. 2025 Nov 20;20:84. doi: 10.1186/s13027-025-00713-8 (PMC12632028; doi:10.1186/s13027-025-00713-8)
Supplement: Supplementary file 2 — Supplementary Material 2 [file 13027_2025_713_MOESM2_ESM.docx]

Supplementary Table 1. Persistence, new infections, and total clearance stratified by baseline HPV-DNA assay

|  |  |  | | | | | | | |  |
| --- | --- | --- | --- | --- | --- | --- | --- | --- | --- | --- |
|  | **Baseline Onclarity positive** | **FU negative for Cobas/HC2 HPV** | **Typing of FU positive for Cobas/HC2 HPV samples** | | | | | | | **Total clearance**  **of baseline infections^#^** |
|  |  |  | **FU positive for Cobas/HC2 HPV** | **Samples with typing** | | | | | **Missing typing** |  |
|  |  |  |  | **Persistence** | | **Only new infections** | | **Negative for typing** |  |  |
|  | **n** | **n (% on total samples - a)** | **n (% on total samples)** | **n (% on positive and typed)** | **n of CIN3+*** | **n (% on positive and typed - b)** | **n of CIN3+*(%)** | **n (% on positive and typed - c)** | **n** | **%** |
|  |  |  |  |  | **(%)** |  |  |  |  |  |
| **Cobas 4800** |  |  |  |  |  |  |  |  |  |  |
| baseline positive | 782 | 300 (38.4) | 482 (61.6) | 146 (78.9) | 0 (0.0) | 19 (10.3) | 0 (0.0) | 20 (10.8) | 297 | 51.4 |
| Single channel | 610 | 259 (42.5) | 351 (57.5) | 98 (75.4) | 0 (0.0) | 16 (12.3) | 0 (0.0) | 16 (12.3) | 221 | 56.6 |
| Multichannel | 172 | 41 (23.8) | 131 (76.2) | 48 (87.3) | 0 (0.0) | 3 (5.5) | 0 (0.0) | 4 (7.3) | 76 | 33.5 |
| **HC2** |  |  |  |  |  |  |  |  |  |  |
| Baseline positive | 758 | 289 (38.1) | 469 (61.9) | 342 (79.9) | 11 (3.2) | 49 (11.4) | 0 (0.0) | 37 (8.6) | 41 | 50.6 |
| Single channel | 609 | 250 (41.1) | 359 (58.9) | 247 (77.7) | 10 (4.0) | 43 (13.5) | 0 (0.0) | 28 (8.8) | 41 | 54.2 |
| Multichannel | 149 | 39 (26.2) | 110 (73.8) | 95 (86.4) | 1 (1.1) | 6 (5.5) | 0 (0.0) | 9 (8.2) | 0 | 36.2 |

^#^ Total clearance includes women who at 1-year retesting were negative for Cobas/HC2 and those who tested negative for Onclarity for the channels that were positive at baseline : a+(1-a)*(b+c)

* Among the CIN3+ cases, no cancer was diagnosed

Supplementary Table 2. Distribution of CIN2+ and CIN3+^§^ between persistent and new infection cases, overall and by baseline cytology, p16/ki67, E6/E7 mRNA, and Onclarity valuable results

|  | **Persistence** | |  | **Only new infections** | |  |
| --- | --- | --- | --- | --- | --- | --- |
|  | **n** | **n of CIN2+ (%)** | **n of CIN3+ (%)** | **n** | **n of CIN2+ (%)** | **n of CIN3+ (%)** |
| **Overall** |  |  |  |  |  |  |
| Single channel | 345 | 17 (4.9) | 10 (2.9) | 59 | 3 (5.1) | 0 (0.0) |
| Multichannel | 143 | 3 (2.1) | 1 (0.7) | 9 | 0 (0.0) | 0 (0.0) |
| **Cytology*** |  |  |  |  |  |  |
| **Negative** |  |  |  |  |  |  |
| Single channel | 286 | 14 (4.9) | 8 (2.8) | 45 | 3 (6.7) | 0 (0.0) |
| Multichannel | 97 | 3 (3.1) | 1 (1.0) | 5 | 0 (0.0) | 0 (0.0) |
| **Positive** |  |  |  |  |  |  |
| Single channel | 56 | 3 (5.4) | 2 (3.6) | 14 | 0 (0.0) | 0 (0.0) |
| Multichannel | 45 | 0 (0.0) | 0 (0.0) | 4 | 0 (0.0) | 0 (0.0) |
| **E6/E7 mRNA*** |  |  |  |  |  |  |
| **Negative** |  |  |  |  |  |  |
| Single channel | 60 | 1 (1.7) | 1 (1.7) | 13 | 0 (0.0) | 0 (0.0) |
| Multichannel | 3 | 0 (0.0) | 0 (0.0) | 0 | 0 (0.0) | 0 (0.0) |
| **Positive** |  |  |  |  |  |  |
| Single channel | 284 | 16 (5.6) | 9 (3.2) | 45 | 3 (6.7) | 0 (0.0) |
| Multichannel | 140 | 3 (2.1) | 1 (0.7) | 9 | 0 (0.0) | 0 (0.0) |
| **p16/ki67*** |  |  |  |  |  |  |
| **Negative** |  |  |  |  |  |  |
| Single channel | 213 | 6 (2.8) | 2 (0.9) | 43 | 1 (2.3) | 0 (0.0) |
| Multichannel | 70 | 2 (2.9) | 1 (1.4) | 7 | 0 (0.0) | 0 (0.0) |
| **Positive** |  |  |  |  |  |  |
| Single channel | 117 | 11 (9.4) | 8 (6.8) | 14 | 2 (14.3) | 0 (0.0) |
| Multichannel | 65 | 1 (1.5) | 0 (0.0) | 2 | 0 (0.0) | 0 (0.0) |
| **Channel positivity** |  |  |  |  |  |  |
| **Single channel** |  |  |  |  |  |  |
| 16 | 70 | 6 (8.6) | 4 (5.7) | 9 | 1 (11.1) | 0 (0.0) |
| 18 | 16 | 1 (6.3) | 0 (0.0) | 4 | 0 (0.0) | 0 (0.0) |
| 45 | 15 | 0 (0.0) | 0 (0.0) | 2 | 0 (0.0) | 0 (0.0) |
| 33/58 | 32 | 1 (3.1) | 1 (3.1) | 3 | 0 (0.0) | 0 (0.0) |
| 31 | 66 | 7 (10.6) | 3 (4.5) | 11 | 0 (0.0) | 0 (0.0) |
| 56/59/66 | 75 | 1 (1.3) | 1 (1.3) | 13 | 1 (7.7) | 0 (0.0) |
| 51 | 19 | 0 (0.0) | 0 (0.0) | 3 | 0 (0.0) | 0 (0.0) |
| 52 | 25 | 0 (0.0) | 0 (0.0) | 7 | 0 (0.0) | 0 (0.0) |
| 35/39/68 | 27 | 1 (3.7) | 1 (3.7) | 7 | 1 (14.3) | 0 (0.0) |
| **Multichannel ^#^** |  |  |  |  |  |  |
| 16/33/58 | 11 | 1 (9.1) | 1 (9.1) | 0 | 0 (0.0) | 0 (0.0) |
| 16/35/39/68 | 21 | 1 (4.8) | 0 (0.0) | 0 | 0 (0.0) | 0 (0.0) |
| 33/58/56/59/66 | 9 | 1 (11.1) | 0 (0.0) | 0 | 0 (0.0) | 0 (0.0) |

^§^ Among the CIN3+ cases, no cancer was diagnosed

*Only samples with a valid test were included: 19 missing or inadequate cytology; 2 missing E6/E7 mRNA; 112 missing or non-valuable P16/ki67

^#^Only the multichannel findings in which CIN2+ lesions were found are reported

Supplementary Table 3. Persistence and new infections stratified by age, cytology, p16/ki67, E6/E7 mRNA results and the number of positive channels

|  | **Baseline Onclarity positive** | **FU negative for Cobas/HC2 HPV** | **Typing of follow-up positive for Cobas/HC2 HPV samples** | | | | |
| --- | --- | --- | --- | --- | --- | --- | --- |
|  |  |  | **FU Positive for Cobas/HC2 HPV** | **Samples with typing** | | | **Missing typing** |
|  |  |  |  | **Persistence** | **Only new infections** | **Negative for typing** |  |
| **Total** | **1540** | **589** | **951** | **488** | **68** | **57** | **338** |
| **Multiple infections** | | | | | | | |
| Single channel | 1219 | 509 | 710 | 345 | 59 | 44 | 262 |
| Multichannel | 321 | 80 | 241 | 143 | 9 | 13 | 76 |
| **Age <40** | | | | | | | |
| Single channel | 344 | 147 | 197 | 96 | 30 | 18 | 53 |
| Multichannel | 103 | 31 | 72 | 48 | 3 | 5 | 16 |
| **Age 40-50** | | | | | | | |
| Single channel | 541 | 234 | 307 | 157 | 21 | 16 | 113 |
| Multichannel | 129 | 31 | 98 | 57 | 3 | 3 | 35 |
| **Age >50** | | | | | | | |
| Single channel | 334 | 128 | 206 | 92 | 8 | 10 | 96 |
| Multichannel | 89 | 18 | 71 | 38 | 3 | 5 | 25 |
| **Cytology*** |  |  |  |  |  |  |  |
| **Negative** |  |  |  |  |  |  |  |
| Single channel | 952 | 401 | 551 | 286 | 45 | 33 | 187 |
| Multichannel | 211 | 53 | 158 | 97 | 5 | 6 | 50 |
| **Positive** |  |  |  |  |  |  |  |
| Single channel | 250 | 100 | 150 | 56 | 14 | 10 | 70 |
| Multichannel | 108 | 27 | 81 | 45 | 4 | 7 | 25 |
| **E6/E7 mRNA*** |  |  |  |  |  |  |  |
| **Negative** |  |  |  |  |  |  |  |
| Single channel | 276 | 135 | 141 | 60 | 13 | 16 | 52 |
| Multichannel | 19 | 8 | 11 | 3 | 0 | 2 | 6 |
| **Positive** |  |  |  |  |  |  |  |
| Single channel | 941 | 374 | 567 | 284 | 45 | 28 | 210 |
| Multichannel | 302 | 72 | 230 | 140 | 9 | 11 | 70 |
| **p16/ki67*** |  |  |  |  |  |  |  |
| **Negative** |  |  |  |  |  |  |  |
| Single channel | 819 | 358 | 461 | 213 | 43 | 26 | 179 |
| Multichannel | 172 | 48 | 124 | 70 | 7 | 8 | 39 |
| **Positive** |  |  |  |  |  |  |  |
| Single channel | 310 | 102 | 208 | 117 | 14 | 13 | 64 |
| Multichannel | 127 | 22 | 105 | 65 | 2 | 4 | 34 |

*Only samples with a valid test result were included: 19 missing or inadequate cytology; 2 missing E6/E7 mRNA; 112 missing or non-valuable p16/ki67
